# Validation of a fall rate prediction model for community-dwelling older adults: a combined analysis of three cohorts with 1850 participants

**DOI:** 10.1186/s12877-024-04811-x

**Published:** 2024-03-27

**Authors:** Christina Wapp, Anne-Gabrielle Mittaz Hager, Toni Rikkonen, Roger Hilfiker, Emmanuel Biver, Serge Ferrari, Heikki Kröger, Marcel Zwahlen, Philippe Zysset

**Affiliations:** 1 ARTORG Center for Biomedical Engineering Sciences, University of Bern, Freiburgstrasse 3, Bern, CH - 3010 Switzerland; 2 Department of Physiotherapy, School of Health Sciences, University of Applied Sciences and Arts Western Switzerland, Leukerbad, Switzerland; 3 Kuopio Musculoskeletal Research Unit, University of Eastern Finland, Kuopio, Finland; 4 Physiotherapy Tschopp & Hilfiker, Valais, Switzerland; 5 Division of Bone Diseases, Department of Medicine, Geneva University Hospitals and Faculty of Medicine, University of Geneva, Geneva, Switzerland; 6 Department of Orthopaedics, Traumatology and Hand Surgery, Kuopio University Hospital, Kuopio, Finland; 7 Institute for Social and Preventive Medicine, University of Bern, Bern, Switzerland

**Keywords:** Falls, Fragility fractures, Older adults, Model validation, Count regression

## Abstract

**Background:**

Fragility fractures in older adults are often caused by fall events. The estimation of an expected fall rate might improve the identification of individuals at risk of fragility fractures and improve fracture prediction.

**Methods:**

A combined analysis of three previously developed fall rate models using individual participant data (*n* = 1850) was conducted using the methodology of a two-stage meta-analysis to derive an overall model. These previously developed models included the fall history as a predictor recorded as the number of experienced falls within 12 months, treated as a factor variable with the levels 0, 1, 2, 3, 4 and ≥ 5 falls. In the first stage, negative binomial regression models for every cohort were fit. In the second stage, the coefficients were compared and used to derive overall coefficients with a random effect meta-analysis. Additionally, external validation was performed by applying the three data sets to the models derived in the first stage.

**Results:**

The coefficient estimates for the prior number of falls were consistent among the three studies. Higgin’s *I*^2^ as heterogeneity measure ranged from 0 to 55.39%. The overall coefficient estimates indicated that the expected fall rate increases with an increasing number of previous falls. External model validation revealed that the prediction errors for the data sets were independent of the model to which they were applied.

**Conclusion:**

This analysis suggests that the fall history treated as a factor variable is a robust predictor of estimating future falls among different cohorts.

**Supplementary Information:**

The online version contains supplementary material available at 10.1186/s12877-024-04811-x.

## Introduction

Falls and fragility fractures are closely associated in older adults. While around one out of three individuals aged 65 years and older fall yearly, a substantial number of those events result in injuries [1]. The incidence of fall-related fractures increases with age, especially for women after 50 [2]. The fact that falls play an important role in fracture prediction is increasingly recognised lately. A meta-analysis using the MrOS study showed that the number of prior falls predicted fractures independently of FRAX [3]. Furthermore, in the latest update of FRAX, the so-called FRAXplus, the history of falls is now included as a risk factor for fractures [4]. In a review paper, Komisar and Robinovitch summarised the relationship between fall biomechanics and fracture risk for distinct fracture sites [5]. Especially hip fractures are almost exclusively caused by falls [6]. Along with reduced bone strength, the risk of a fall and the inability to counteract such a fall event can lead to a fracture. Accordingly, individuals with a higher fall frequency and severity are simultaneously exposed to an increased fracture risk. Subsequently, predicting how often a person is likely to fall could help identify individuals at risk for fragility fractures.

However, the focus of fall risk assessments presented in the literature is on identifying people at risk of falling, not on predicting the number of expected falls. This becomes evident when reviewing the literature on this topic [7–9]. As an alternative to binary logistic regression that assesses the risk of falling as a probability between 0 and 1, count regression models allow the prediction of rate ratios and thus, the calculation of the expected number of falls within a time period [10]. However, only a few studies analysing the risk of falling in terms of fall rates have been published [11, 12]. For example, a study conducted by Gade et al. developed the fall rate prediction model for community-dwelling older adults by fitting a Poisson regression and using the least absolute shrinkage and selection operator penalization for variable selection [11].

Similarly, we analysed three independent cohorts investigating aspects of the risk of falling in community-dwelling older adults and developed fall rate prediction models in previous work. The three cohorts are the Geneva Retirees Cohort (GERICO) [13], the Swiss CHEF Trial (SCT) [14], and the Kuopio Fall Prevention Study (KFPS) [15, 16]. Fall rate prediction models were developed using a count regression modelling approach, and two of the three analyses have been published previously [17, 18]. In short, the results showed that the history of falls measured as the number of prior falls within 12 months before the study examination was the best predictor for future falls in all three cohorts [17, 18]. Furthermore, we showed the importance of how the information about the fall history is treated as a predictor. In most prediction models, this information is included as binary information (yes/no) for fallers in general or recurrent fallers [19]. However, valuable predictive information gets lost by condensing the prior number of falls into a binary variable. When comparing the rate ratio for an individual who experienced 5 falls, we found the model coefficient estimate to be 4 times higher when the information is treated as a factor variable compared to a binary variable [17].

Against this background, and with the further goal of improving fragility fracture prediction by including information on falls, this study aimed to compare models for predicting fall rates that included the history of falls as a categorical predictor. We used the methodology of a two-stage meta-analysis to compare the model coefficients and suggest an overall prediction model. Additionally, we performed an external validation between the three previously developed models.

### Methods

#### Cohorts and data

The two main criteria for inclusion in this combined analysis were that the data was analysed using a count regression method and that the predictor history of falls was treated as a factor variable. Apart from the three models that we developed previously, we are unaware of any other studies meeting those criteria.

**Fig. 1:**
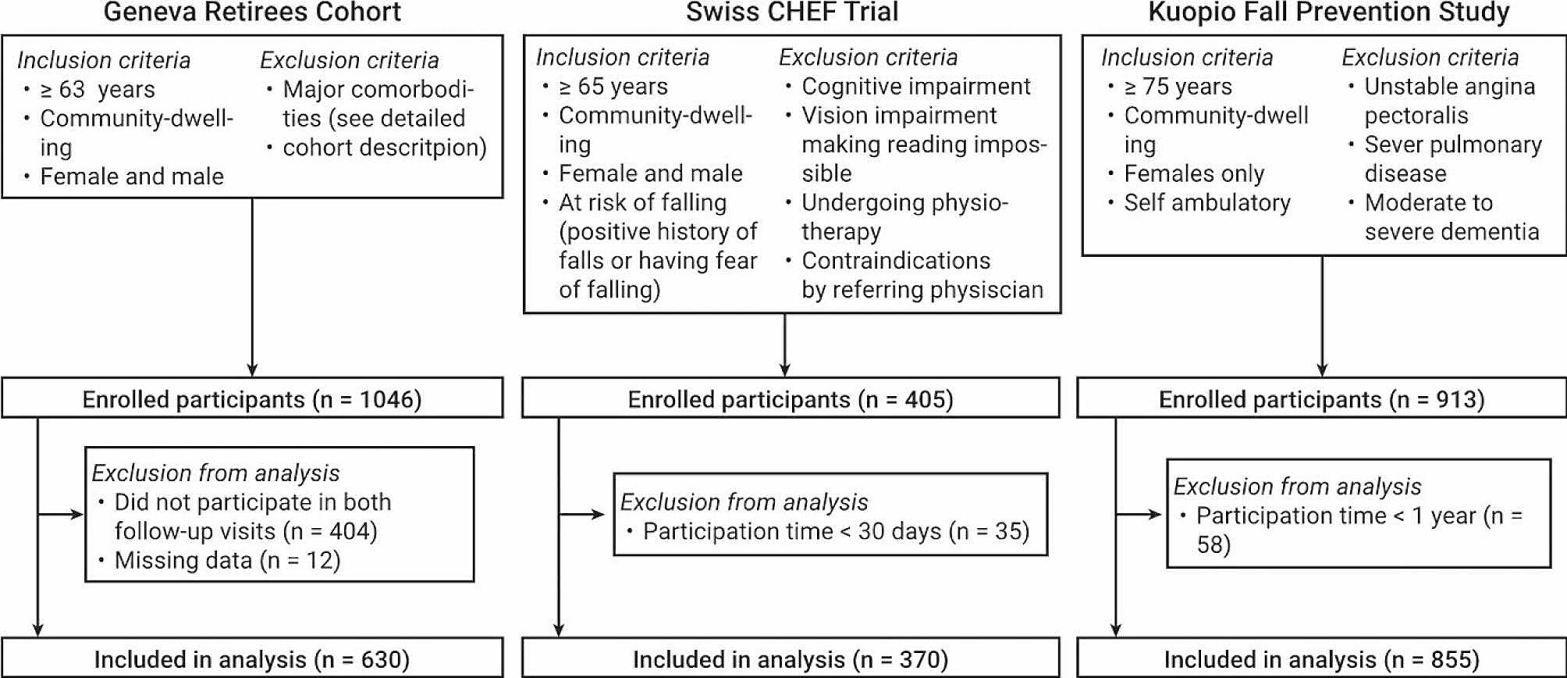
Flow of participants in the three cohorts

Individual participant data were available from the original data sets of all cohorts. The analysis and development of the GERICO and SCT prediction models have been published previously [17, 18], and the analysis of the KFPS is available in the supplementary material. A list of all predictors investigated in the prior analyses can be found in the supplementary material, eTable 1. The flow of participants and the inclusion and exclusion criteria for the cohorts and this analysis are presented in Fig. 1.

**Table 1:** Comparison of the trial designs and cohort characteristics

	GERICO	SCT	KFPS
Country	Geneva, Switzerland	Valais, Switzerland	Kuopio, Finland
Study design	Prospective observational trial	Prospective RCT	Prospective RCT
Setting	Community-dwelling older adults	Community-dwelling older adults	Community-dwelling older adults
No. of participants enrolled in study	1046	405	913
No. of participants included in analysis	630	370	855
Sex (male/female)	126/504	100/270	0/855
Mean age (SD) [years]	67.9 (1.6)	78.7 (6.8)	76.5 (3.2)
Previous falls during 12 months			
Number	646	537	627
Mean	1.03	1.45	0.73
Reporting	Self-reported retrospective	Self-reported retrospective	Self-reported retrospective
Incidence falls			
Number	439	371	755
Mean^a^	0.69	1.30	0.83
Reporting	Self-reported, retrospective	Self-reported with monthly falls calendar, prospective	Biweekly SMS and phone-calls, prospective

*Abbreviations* GERICO = Geneva Retirees Cohort; KFPS = Kuopio Fall Prevention Study; RCT = randomised controlled trial; SCT = Swiss CHEF Trial; SD = standard deviation. ^a^per person year

#### Geneva Retirees Cohort

The Geneva Retirees Cohort (GERICO) is a prospective observational study conducted between 2008 and 2018 around Geneva, Switzerland. It aimed to investigate the risk factors for fracture and fall prediction in community-dwelling older adults. Participants were enrolled in the study between 2008 and 2011 and invited for a baseline examination. Two follow-up visits were conducted after 4 and 8 years each. The study was described previously, and the trial was registered under www.isrctn.com/ISRCTN11865958.

##### Participants

Participants were community-dwelling older adults of both sexes, with a mean age of 67.9 years (1.6 standard deviation (SD), range 64.6–71.8) at follow-up visit 1 and living in rural or urban areas around Geneva.

##### Exclusion criteria

Participants were excluded if they suffered from major comorbidities, particularly cancer treated within the last 5 years, chronic renal failure, liver or lung disease, corticosteroid therapy, primary hyperparathyroidism, Paget disease of bone, malabsorption or any neurological or a musculoskeletal condition affecting bone health.

##### Variables of interest

Fall risk-related variables of importance for the fall rate model development were mainly recorded during the two follow-up visits. These included age, body mass index, short physical performance battery, hand grip strength, one-legged stance test, activity level, Charlson’s comorbidity index, the number of comorbidities, and the number of medication.

##### Falls

A fall was defined as an event resulting in unintentionally coming to rest on the ground, floor or any lower levels. Falls were assessed retrospectively at the two follow-up visits by asking whether any falls occurred during the last 12 months.

#### Swiss CHEF Trial

The Swiss CHEF Trial (SCT) is a randomised controlled trial investigating three home-based exercise programs for fall prevention in community-dwelling older adults. The study was conducted between 2016 and 2022 in Switzerland. The study was described previously, and the trial was registered under https://clinicaltrials.gov/study/NCT02926105.

##### Participants

Participants enrolled in the study were community-dwelling older adults of both sexes with a mean age of 78.7 years (6.8 SD, range 65–100), who fell at least once in the previous 12 months or were afraid of falling (FES-I score of at least 20 points).

##### Exclusion criteria

Exclusion criteria were severe visual impairment, cognitive impairment (< 24 points on the Mini Mental State Examination), physiotherapeutic treatment with balance training, or contraindication by the referring physician.

##### Variables of interest

Variables such as demographic characteristics, history of falls in the previous 12 months, fear of falling, physical performance tests, health state and quality of life were assessed at a baseline examination.

##### Intervention

Participants were divided into three intervention groups using block randomisation. The intervention programs were (1) a newly developed intervention program called Test&Exercise, (2) the Otago exercise program as a reference group [20], and (3) an intervention representing usual care in Switzerland as control group. This consisted of a small booklet with 12 exercises for balance and strength training, as a control group. The intervention lasted 6 months, with another 6 months of follow-up afterwards. After 6 and 12 months, the baseline examinations were remeasured.

##### Falls

A fall was defined as an unexpected event in which the participant comes to rest on the ground, floor, or lower level, with or without injury. Incident falls were prospectively self-reported with a monthly fall calendar during the 12 months of intervention and follow-up. History of falls was assessed at baseline by asking how many falls occurred during the previous 12 months.

#### Kuopio Fall Prevention Study

The Kuopio Fall Prevention Study (KFPS) is a 2-year randomised controlled trial to estimate the effect of a fall prevention exercise program in community-dwelling older women in Kuopio, Finland [16]. The trial was launched in 2016. The study was registered under  https://clinicaltrials.gov/study/NCT02665169, and the detailed trial protocol was published in BMC Geriatrics [14].

##### Participants

Participants enrolled were female only, had a mean age of 76.5 years (SD 3.2, range 71.2–84.8), were living around the City of Kuopio, were able to attend exercise sessions twice a week and were in an adequate health state (self-ambulatory, no unstable angina pectoris, no severe pulmonary disease, no moderate to sever dementia).

##### Exclustion criteria

Individuals living in institutional long-term care homes were excluded from the study.

##### Variables of interest

These included functional tests, social well-being, cognitive performance, sarcopenia and frailty measurements.

##### Intervention

After baseline examination, participants were divided into intervention and control groups using block randomisation. The intervention included initial 6 months of supervised exercise including the free acces to municipal exercise facilities, another 6 months of unsupervised use and free access to exercise facilities, and following 12 months of low-cost access to exercise facilities. The control group also had low-cost access to exercise facilities without supervision for 24 months. Variables of interest were assessed at the baseline, at 12 months and 24 months.

##### Falls

A fall was defined according to the WHO International ICD diagnosis code. Falls from the same level, on stairs, and from height were included. Incident falls were recorded biweekly via SMS, and in case of positive reports assessed with telephone interviews. History of falls was assessed at baseline by asking how many falls occurred during the previous 12 months [15].

#### Participants included in the meta-analysis

Inclusion criteria for the meta-analysis were defined for every cohort separately. Participants of the GERICO cohort had to have participated in the two follow-up visits from the study. For the SCT analysis, the participants were required to remain enrolled for at least one month after the baseline examination. For the KFPS study, participants had to have participated for at least one year. The flow of participants with inclusion and exclusion criteria for every cohort and this analysis are presented in Fig. 1. A completed case analysis was conducted.

#### Statistical analysis

##### Outcome

The outcome variable was the number of incident falls. For SCT and KFPS, this referred to the reported number of falls during intervention and follow-up. For GERICO, the outcome was the number of falls reported at the second follow-up visit.

##### Predictors

The final models of all three cohorts included the history of falls measured as the prior number of falls during 12 months as a predictor. In the GERICO and KFPS study, it was the only predictor included in the suggested models. In the SCT model, fear of falling measured with FES-I was the only additional predictor. Since fear of falling was not assessed in the other two cohorts, it was not included in this analysis. In the analysis of the SCT study, we showed that the number of prior falls is best treated as a factor variable with levels 0, 1, 2, 3, 4 and ≥ 5, in contrast to using it as binary information (previous falls yes vs. no) or a continuous variable [17]. Therefore, the number of prior falls was introduced as a factor variable with those six levels. No falls was defined as the reference category in all three cohorts.

##### Combined analysis

The combined analysis was performed using the methodology of a two-stage meta-analysis as described by Burke et al. [21]. In the first stage, the prediction models were fit separately for every data set with negative binomial regression models, resulting in a coefficient estimate for every level of the factor variable. The SCT model included an offset because not all participants were followed up for 12 months.

In the second stage, the three resulting coefficient estimates and standard deviations were meta-analysed for each factor level and the dispersion parameter $\theta$. A random effect model with inverse variance weighting was fitted. ${\tau }^{2}$ was estimated with the restricted maximum likelihood estimator. Higgin’s *I*^*2*^ was computed to investigate the percentage of variance attributable to the study heterogeneity among the true effects.

##### Model validation and calibration

The apparent absolute mean prediction error for the three first-stage models was calculated. In addition, the three models were externally validated by calculating the prediction error for unseen data, e.g. using the GERICO model, the prediction error was derived for the SCT and the KFPS data set. The prediction error of the overall model derived with the combined analysis was calculated with all three cohorts. The method for calibration-in-the-large was adapted from Crowson et al. [22], suggesting a regression model-based framework for calibrating survival data. The following steps were performed on the link scale: (1) fit the new data to the existing model, resulting in a linear predictor $$ {p}_{0}$$ (2) fit a new negative binomial regression model with the outcome variable from the new data set $$ {outcome}_{new}$$ and using the linear predictor $$ {p}_{0}$$ as an offset, (3) use the intercept $$ {\alpha }_{new}$$ derived from the model fitted in step 2 to update $$ {p}_{0}$$ such that the updated prediction $$ {p}_{1}$$ is derived as $$ {p}_{1}={a}_{new}+ {p}_{0}$$. $$ {\alpha }_{new}$$ is referred to as the calibration-in-the-large or the recalibration constant. A detailed example of the R code can be found in the supplementary material. Calibration was assessed by plotting the expected versus the observed number of falls in form of a rootogram [23, 24].

##### Statistical program

All statistical analysis was conducted with R Studio Version 4.2.2. For the meta-analysis, the package “metafor” was used [25].

### Results

#### Study characteristics

All three studies were prospective trials including community-dwelling older adults. While the SCT and the KFPS were randomised controlled trials to investigate new fall prevention interventions, the GERICO study was an observational study. The number of participants enrolled in the GERICO, SCT, and KFPS were 1046, 405, and 913, respectively. Of these, 642, 370, and 855, respectively, fulfilled the inclusion criteria for the analysis. Twelve participants had missing fall data in the GERICO study, resulting in 630 participants included in the analysis. The GERICO and SCT cohorts included both sexes, with mostly females (GERICO: 80%, SCT: 73%). Only women participated in the KFPS. The mean age was 67.9 years for GERICO, 78.7 years for SCT, and 76.5 years for KFPS. In total, 1810 falls were reported before the baseline examination, and 1565 falls after the baseline examination. For the GERICO trial, the mean number of falls during the 12 months before the follow-up visit 1 was 1.03 and decreased to 0.69 falls during the 12 months before the follow-up visit 2. In the SCT, the mean number of reported falls during 12 months before the baseline examination was 1.45, and 1.30 falls per person-year were reported for the year following the baseline examination. In the KFPS, 0.73 falls per person have been reported before baseline examination, and 0.83 in the subsequent 12 months. All results comparing the trial and cohort characteristics are presented in Table 1.

#### Combined analysis

**Fig. 2:**
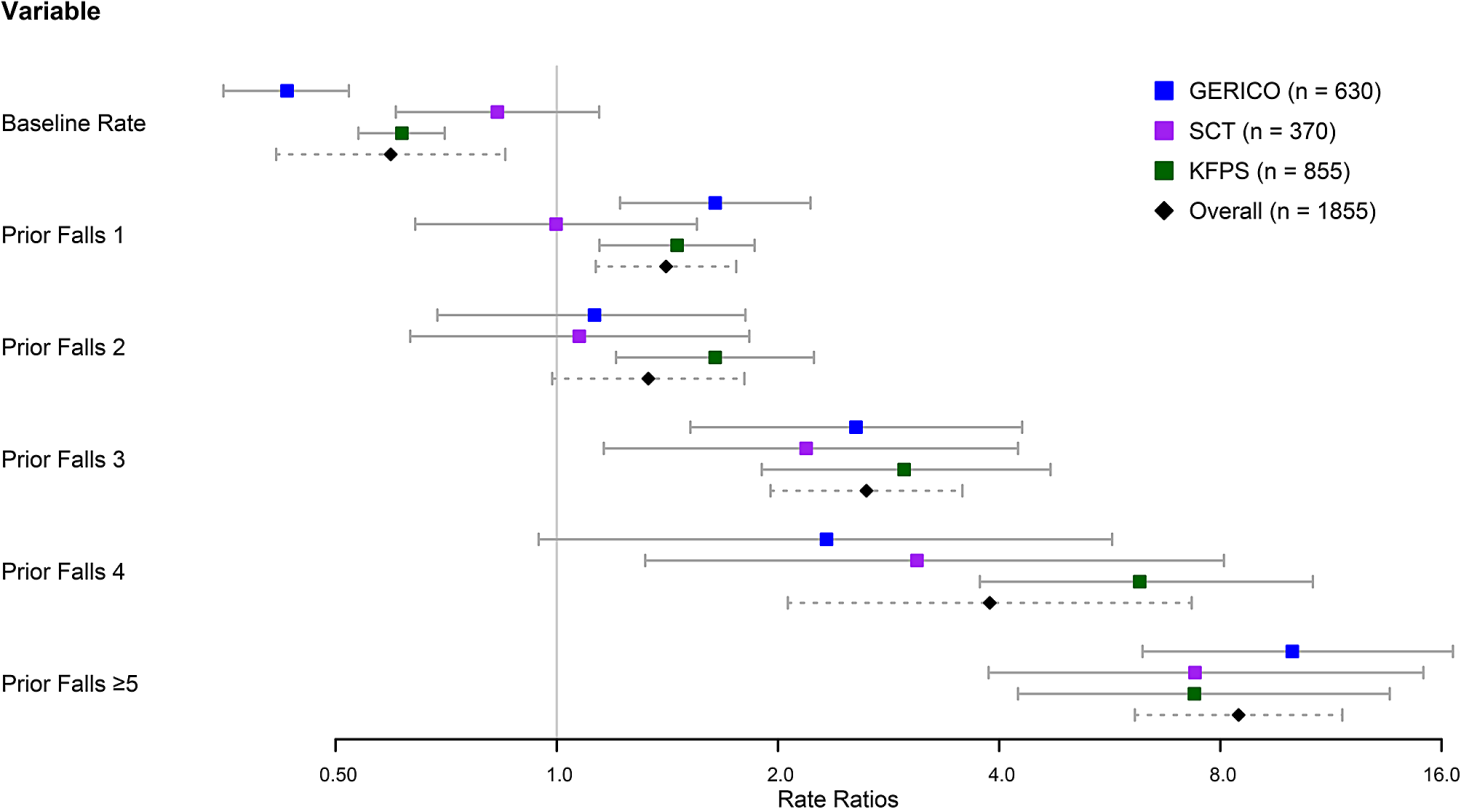
Baseline rate and rate ratios for the model coefficients with 95% confidence intervals

The results of the three models fitted in the first stage and of the random effect models derived in the second stage are shown in a forest plot in Fig. 2; Table 2. The heterogeneity measures for the coefficients are also presented Table 2.

**Table 2:** Rate ratios with 95% confidence interval and heterogeneity measures for all models

	Rate ratios (95% CI)				Heterogeneity	
	GERICO *n* = 630	SCT *n* = 370	KFPS *n* = 855	Overall *n* = 1855	${\varvec{\tau }}^{2}$	${\varvec{I}}^{2}$ (%)
Baseline rate (per year)	0.43 (0.35 to 0.52)	0.83 (0.61 to 1.14)	0.61 (0.54 to 0.70)	0.59 (0.41 to 0.85)	0.088	89.24
Prior falls 1	1.64 (1.22 to 2.21)	1.00 (0.64 to 1.54)	1.46 (1.15 to 1.87)	1.41 (1.13 to 1.76)	0.013	33.50
Prior falls 2	1.13 (0.70 to 1.82)	1.07 (0.63 to 1.82)	1.65 (1.21 to 2.25)	1.33 (0.98 to 1.81)	0.026	34.55
Prior falls 3	2.55 (1.52 to 4.29)	2.18 (1.15 to 4.15)	2.98 (1.90 to 4.68)	2.64 (1.96 to 3.57)	0.000	00.00
Prior falls 4	2.33 (0.96 to 5.65)	3.09 (1.26 to 7.58)	6.24 (3.71 to 10.48)	3.89 (2.06 to 7.34)	0.169	53.19
Prior falls ≥ 5	10.02 (6.17 to 16.27)	7.39 (3.77 to 14.46)	7.40 (4.15 to 13.20)	8.48 (6.13 to 11.74)	0.000	00.00
$\theta$	1.06 (0.71 to 1.42)	0.66 (0.44 to 0.87)	1.18 (0.86 to 1.50)	0.94 (0.61 to 1.27)	0.25	73.85

*Abbreviations* n = number of participants; $$ {I}^{2}$$ = Higgin’s $$ {I}^{2}$$; CI = confidence interval; $$ \theta $$ = dispersion parameter; GERICO = Geneva Retirees Cohort; SCT = Swiss CHEF Trial; KFPS = Kuopio Fall Prevention Study

The baseline rate or intercept varied among the three cohorts (GERICO: 0.43 [95% CI from 0.35 to 0.52]; SCT: 0.83 [95% CI from 0.61 to 1.14]; KFPS: 0.61 [95% CI from 0.54 to 0.79]). The overall estimate for the baseline rate derived with the random effect model was 0.59 (95% CI from 0.41 to 0.85) and showed a high heterogeneity ($$ {\tau }^{2}$$:0.088, $$ {I}^{2}$$: 89.24%). The rate ratios for one prior fall (GERICO: 1.64 [95% CI from 1.22 to 2.21]; SCT: 1.00 [95% CI from 0.64 to 1.54]; KFPS: 1.46 [95% CI from 1.15 to 1.87]) were in a comparable magnitude as for two prior falls (GERICO: 1.13 [95% CI from 0.70 to 1.82]; SCT: 1.07 [95% CI from 0.63 to 1.82]; KFPS: 1.65 [95% CI from 1.21 to 2.25]). Accordingly, the overall estimates were 1.41 (95% CI from 1.13 to 1.76) for one prior fall and 1.33 (95% CI from 0.98 to 1.81) for two prior falls. Heterogeneity was also comparable and lower for the baseline rate (one prior fall: $$ {\tau }^{2}$$: 0.013, $$ {I}^{2}$$: 33.50%; two prior falls $$ {\tau }^{2}$$: 0.026 $$ {I}^{2}$$: 34.55%). The rate ratios for three prior falls increased similarly in all three studies (GERICO: 2.55 [95% CI from 1.52 to 4.29]; SCT: 2.18 [95% CI from 1.15 to 4.15]; KFPS: 2.98 [95% CI from 1.90 to 4.68]), resulting in an overall effect estimate of 2.64 (95% CI from 1.96 to 3.57). The two heterogeneity measures $$ {\tau }^{2}$$ and $$ {I}^{2}$$ were equal to zero. The rate ratios for four prior falls were more heterogenous, with the highest estimate for the KFPS (GERICO: 2.33 [95%CI from 0.96 to 5.65]; SCT: 3.09 [95% CI from 1.26 to 7.58]; KFPS: 6.24 [95% CI from 3.71 to 10.48]). The overall estimate was 3.89 (95% CI from 2.06 to 7.34), with the heterogeneity reflected in the corresponding measures ($$ {\tau }^{2}$$: 0.169, $$ {I}^{2}$$: 53.19%). The highest estimates were reached for five or more prior falls (GERICO: 10.02 [95% CI from 6.17 to 16.27]; SCT: 7.39 [95% CI from 3.77 to 14.46]; KFPS: 7.40 [95% CI from 4.15 to 13.20]) resulting in an overall effect estimate of 8.48 (95% CI from 6.13 to 11.74) with no heterogeneity present ($$ {\tau }^{2}$$: 0.000, $$ {I}^{2}$$: 0.00%).

#### Model validation and calibration

**Fig. 3:**
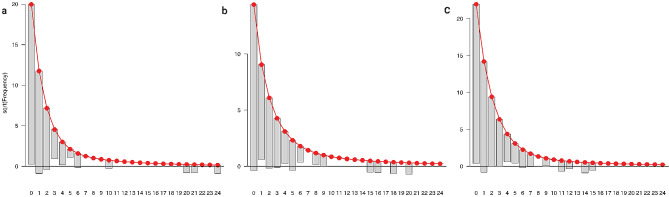
Hanging rootograms as marginal calibration diagrams for (**a**) the GERICO data, (**b**) the SCT data and (**c**) the KFPS data applied to the overall model showing the deviation between the actual (grey bars) and predicted (red line) number of individuals per fall number category

The apparent mean absolute prediction error was highest for the SCT followed by KFPS and GERICO (GERICO: 0.82; SCT; 1.16; KFPS: 0.92). For the external model validation, the mean absolute prediction error for the GERICO data set was comparable to the apparent error when applied to the other three models, (SCT model: 0.82; KFPS: 0.81 model; Overall model: 0.81). Similar results were found for the SCT data set (GERICO model: 1.19; KFPS model: 1.14; Overall model: 1.15), and the KFPS data set (SCT model: 0.94; KFPS model: 0.92; Overall model: 0.92). These results indicate that the models here are not prone to overfitting and hardly any bias. In addition, the method used for recalibration can catch the baseline rate of the cohorts. The result of the model validation and the recalibration constant between the models are summarised in the supplementary materials in eTable 2. Marginal calibration plots for the three data sets applied to the overall model in the form of a hanging rootogram are presented in Fig. 3. The bars represent the observed frequency per fall number category, while the red curve shows the expected frequency. Deviations between expected and observed can be seen when focusing on the x-axis: whereas overshooting into the negative y-values means underestimation, floating bars not reaching the x-axis indicate overestimation of the expected frequency estimated by the prediction model. The diagrams show that the overall model is well calibrated, especially in the range of low fall numbers. The biggest difference can be found for high-frequency fallers, such as 20 falls or more. The rootograms for the other combinations of models and data sets (e.g., SCT data applied to the GERICO model) can be found in the supplementary materials in eFigure 1.

### Discussion

This analysis compared three fall rate prediction models that were previously developed in independent cohorts and derived overall model coefficients using the methodology of a two-stage meta-analysis. Additionally, external model validation including model recalibration was performed. We found that the coefficient estimates among the three models were reasonably consistent, which was also reflected in heterogeneity measures such as Higgin’s $$ {I}^{2}$$. The heterogeneity seen in the baseline rate can be explained by the different fall incidences in the cohorts. However, such differences can be adjusted for with proper calibration methods, as for example suggested by Crowson et al. [22]. Our findings suggest that the number of prior falls as a factor variable is a robust predictor for future falls in community-dwelling older adults among different cohorts. Further studies and investigations are required to find out whether the model can be transferred to even more different settings, for example, to older adults living in institutions, or the oldest old.

Despite the differences in study design and cohort characteristics, the prediction error for the cohorts was shown to be independent of the model that was used to compute the prediction, indicating that no bias in the first-stage models was present. However, no external validation was done for the overall model. In order to check for bias in the overall model towards the data it was derived with, an unseen dataset is required. When comparing the prediction errors presented in this analysis with literature, only one study comes in quest. The prediction error of the PREFALL model that was derived using a similar development strategy is in the same range as our results [11]. They report a bootstrapped mean absolute error of 0.88 falls per year. Further comparisons with other studies are only possible to a limited extent, as most fall prediction models are based on predicting the fall risk and not the fall rate.

Although it is known that there exists a vast amount of risk factors that are associated with falling, the previously conducted analysis of the three cohorts showed that prior falls were superior in predicting future falls compared to other predictors. Variables such as physical performance tests, age, sex, comorbidities, medication, or quality of life were not improving the predictive accuracy of the models in combination with the history of falls [17, 18]. Fear of falling was the only additional predictor selected with variable selection in the SCT study. However, this information was not recorded in all three studies and could not be considered in this analysis. One reason for the lack of further predictors in the models could be the complexity and multifactorial nature of the fall, which can vary greatly from person to person. While one person may be falling due to the combination of vision impairment and balance problems, another may fall because of a lack of strength and a medication that has a side effect of dizziness. It may not be possible to capture or assess all relevant combinations of risk factors for each person in a statistical model. Hence, the presence of prior falls themselves might be the best reflection of whether an individual is exposed to the relevant combination of risk factors for falling. Nevertheless, this bears the risk that the model cannot properly catch first-time fallers. All individuals without a history of falls have an identical predicted fall rate, which does not reflect reality. Therefore, further risk factors sensitive enough to catch first-time fallers must be identified, even if information about the fall history is available. Once identified, the model proposed here could be updated accordingly.

#### Strength and limitations

A strength of this study is the large number of data points available for this analysis: In total 1855 participants were included in this combined analysis. In addition, individual participant data were accessible, enabling the identical treatment of outcome and predictor variables among the three cohorts and thus the application of a two-stage meta-analysis methodology. Furthermore, the history of falls was recorded as the number of previously experienced falls, providing more detailed information than a dichotomised variable (yes versus no).

This analysis also has some limitations, which mainly concern the study design. First, the SCT and KFPS studies were designed as prospective randomised controlled trials with preventive interventions that potentially impact the observed fall incidence rates. Accordingly, the results could differ compared to purely observational data. However, when comparing with the results from GERICO analysis as an observational data set, such differences were not found. Yet, in the GERICO study, incident falls and history of falls were recorded retrospectively at two time-points four years apart. Four years between the two visits is a long time span in a fall prediction setting. In addition, it has been reported that retrospective reporting can result in deviations of the true fall number [26]. Furthermore, the inclusion criteria for participants of the three studies differed: In the SCT study, participants were the only ones who had to be classified as at risk of falling for enrolment, while participants with major comorbidities were excluded in the GERICO study. This might have led to a different selection of study participants. Next to that, the sex distribution among the participants was not balanced, with a vast majority of female participants. And last, the individuals who participated in these three studies have been enrolled out of self-motivation. It has been reported that such individuals are health-wise better off compared to nonparticipants, resulting in a selection bias and may limit the generalisability of such findings [27].

#### Clinical implications and applicability

As the majority of non-vertebral fragility fractures are the result of a fall, the risk of injury increases directly together with the frequency of falls. Accordingly, the estimate of how many times an individual is going to fall can help improve fracture prediction. However, not only fractures but many other injury types in older adults are a consequence of falls [1]. Therefore, estimating a fall rate might also be beneficial in other fields of injury prevention. The simple question “How many times did you fall in the last 12 months?” would be sufficient to derive the fall rate estimate. This information can be further used or integrated into subsequent models to estimate the risk of an event of interest. We want to stress that asking for the number of falls, and not just whether falls have occurred, is helping to improve prediction accuracy. Furthermore, we suggest that falls should be reported as numbers and not as binary variables in research articles.

To make the model applicable in different geographical settings, calibration considering the differences of fall incidences between regions or countries is required. The method presented here to recalibrate between cohorts [22] showed good performance and is easily implemented.

## Conclusion

We found that the number of previous falls treated as a factor variable is a robust predictor of estimating fall rates among different cohorts. In addition, a proper recalibration can account for variations in fall incidences between different cohorts. Further investigations are required to find predictors that can identify first-time fallers.

### Electronic supplementary material

Below is the link to the electronic supplementary material.

Supplementary Material 1

## Data Availability

R Code files and data will be made available upon reasonable request. Please get in touch with the corresponding author through christina.wapp@unibe.ch.

## Abbreviations

FES-I: Falls Efficacy Scale International
GERICO: Geneva Retirees Cohort
KFPS: Kuopio Fall Prevention Study
RC: Recalibration constant
SCT: Swiss CHEF Trial
SD: Standard deviation
