# Supplementary material for: Validation of a fall rate prediction model for community-dwelling older adults: a combined analysis of three cohorts with 1850 participants
Source: BMC Geriatr. 2024 Mar 27;24:287. doi: 10.1186/s12877-024-04811-x (PMC10967162; doi:10.1186/s12877-024-04811-x)
Supplement: Supplementary file 1 — Supplementary Material 1 [file 12877_2024_4811_MOESM1_ESM.docx]

**Supplementary Material**

1. Development of prediction model for the Kuopio Fall Prevention Study
2. R Code Calibration-in-the-large
3. Supplementary tables and figures

**1. Development of the prediction model for the Kuopio Fall Prevention Study**

**Methods**

***Participants***

Participants were included in the analysis if they completed at least 1 year of follow-up.

***Outcome variable***

The outcome variable was the prospectively reported number of falls during the first 12 months after the baseline examination.

***Predictors***

*General characteristics:* Age; Body mass index; living alone (yes/no)

*History of falls:* Number of falls experienced during the 12 months before the baseline examination

*Fear of falling:* The following question was asked: “Are you afraid of falling today?” Answer options were: no / yes, rarely / yes, sometimes / yes, often / yes, constantly.

*Physical performance tests:* Able to squat to the floor (yes/no); One-legged stance test (at least 3 seconds, maximum 30 seconds); hand grip strength (maximum dominant hand, measured in kg), Timed Up and Go (seconds); body sway (open and closed eyes, normal stance and semi-tandem, measured in mm); isometric leg extension force (left and right leg, two attempts each, measured in Nm).

*Activity and physical performance:* Self-perceived physical condition (very bad / pretty bad / satisfactory / pretty good / very good); Exercise frequency (almost never / 1-3x a months / 1x a week / 2x a week / 3x a week /4x a week / 5x a week / (almost) daily); physical inactive hours per day;

*Health state:* Self-perceived health state (very good / good / mediocre / bad); number of medication;

*Intervention group*: Intervention vs control group

***Processing of predictors***

Age was centred at 65 years. The number of prior falls was treated as a categorical variable with the levels 0, 1, 2, 3, 4 and ≥5 falls. Fear of falling was summarised to three levels (no / yes, rarely + yes, sometimes / yes, often + yes, constant). Body sway was included as the difference between open and closed eyes with normal stance. The self-perceived physical condition was summarised into three levels (very bad + pretty bad / satisfactory / pretty good + very good). Exercise frequency was summarised into four levels (almost never + 1-3x a month / 1-2x a week / 3-4x a week / 5x or more a week). The number of medication was also dichotomized (0-2 / 3-4 / 5-6 / ≥7).

***Missing data***

A completed case analysis was conducted.

***Model fit and variable selection***

A model including all candidate predictors was fit. Variable selection was then conducted with backward elimination using the Bayesian information criteria as a stopping rule.

**Results**

***Participants***

In total, 913 participants were enrolled in the study. Thereof, 855 completed at least one year of follow-up. 38 (4.4%) had missing data and were excluded from the analysis, resulting in 817 participants used for the prediction model development.

***Selected model***

The final model included the prior number of falls as the only predictor.

|  | Incidence rate ratios (95% CI) |
| --- | --- |
| Intercept | 0.62 (0.54 to 0.71) |
| 0 prior falls | *Ref* |
| 1 prior fall | 1.47 (1.15 to 1.88) |
| 2 prior falls | 1.62 (1.19 to 2.23 |
| 3 prior falls | 2.97 (1.90 to 4.65) |
| 4 prior falls | 6.21 (3.71 to 10.40) |
| ≥ prior falls | 7.37 (4.16 to 13.09) |

***Prediction error***

The apparent mean absolute prediction error of the selected model was 0.92.

**2. R Code: Calibration in the large**

# fit the original model for the SCT data

> model_sct <- glm.nb(falls ~ prior_falls + offset(log(participation_time), data = data_sct)

# calculate the linear predictor for the KFPS data with the SCT model

> p0_kfps <- predict(model_sct, newdata = data_kfps, response = “link”)

# fit a new negative binomial model with the falls from KFPS as outcome variable and p0_kfps as offset

> recal_model <- glm.nb(falls ~ offset(p0_kfps), data = kfps)

# use the intercept alpha derived from the recal_model to update the predictions p0_kfps

> p1_kfps <- alpha + p0_kfps

# if wanted, the updated linear predictor can now be transformed to the response scale with exp(), resulting in expected frequencies

> p1_kfps_resp <- exp(p1_kfps)

**3. Supplementary table**

eTable 1. Variables assessed in the different cohorts related to the risk of falling.

| **Variables** | | **GERICO** | **SCT** | **KFPS** |
| --- | --- | --- | --- | --- |
| *General* | |  |  |  |
|  | Age | x | x | x |
|  | Sex | x | x | x |
|  | BMI | x | x | x |
|  | Living area (urban/rural) |  | x |  |
|  | Living alone (yes/no) |  |  | x |
| *Fear of falling* | |  |  |  |
|  | FES-I |  | x |  |
|  | Fear of falling |  | x | x |
| *Physical performance tests* | |  |  |  |
|  | Gait speed | x | x |  |
|  | Five Times Sit-to-Stand | x | x |  |
|  | Balance test | x |  |  |
|  | Hand grip strength | x |  | x |
|  | One Legged Stance Test | x | x | x |
|  | Functional reach test |  | x |  |
|  | Timed Up and Go |  | x | x |
|  | Four Stage Balance Test |  | x |  |
|  | Base of support width |  | x |  |
|  | Body sway |  |  | x |
|  | Able to squat to floor (yes/no) |  |  | x |
|  | Isometric leg extension force |  |  | x |
|  | Physical activity (kcal/day) | x |  |  |
|  | Exercise frequency |  |  | x |
|  | Physical condition (self-perceived) |  |  | x |
| *Health state and comorbidities* | |  |  |  |
|  | Charlson’s Comorbidity Index | x |  |  |
|  | Comorbidity number | x |  |  |
|  | Medication number | x | x | x |
|  | Hearing problems (yes/no) |  | x |  |
|  | Vision impairment (yes/no) |  | x |  |
|  | Walking aid (yes/no) |  | x |  |
|  | Urinary incontinence (yes/no) |  | x |  |
|  | Musculoskeletal disorder (yes/no) |  | x |  |
|  | Neurological disorder (yes/no) |  | x |  |
|  | Perceived pain (range 0 – 100) |  | x |  |
|  | Self-perceived health state |  |  | x |
| *Quality of life* | |  |  |  |
|  | OPQOL-35 |  | x |  |

GERICO = Geneva Retirees Cohort; SCT = Swiss CHEF Trial; KFPS = Kuopio Fall Prevention Study; OPQOL-35 = Older People’s Quality of Life Questionnaire.

eTable 2. Mean absolute prediction error for every data set applied to the different models.

|  |  | *Data used for prediction* | | | | | | | |
| --- | --- | --- | --- | --- | --- | --- | --- | --- | --- |
| *Model* |  | GERICO | |  | SCT | |  | KFPS | |
|  |  | MAE | RC |  | MAE | RC |  | MAE | RC |
| GERICO |  | 0.82 | - |  | 1.19 | 0.47 |  | 0.94 | 0.41 |
| SCT |  | 0.82 | -0.47 |  | 1.16 | - |  | 0.94 | -0.12 |
| KFPS |  | 0.81 | -0.35 |  | 1.14 | 0.06 |  | 0.92 | - |
| Overall |  | 0.81 | -0.29 |  | 1.15 | 0.15 |  | 0.92 | 0.09 |

Abbreviations. GERICO = Geneva Retirees Cohort; SCT = Swiss CHEF Trial; KFPS = Kuopio Fall Prevention Study; MAE = mean absolute error; RC = recalibration constant

eFigure1. Hanging rootograms as marginal calibration showing the deviation between the actual (grey bars) and predicted (red line) number of individuals per fall number category. The x-axis represents models, while the y-axis stands for data sets, meaning, e.g. the top right plot is the calibration diagram for the KFPS data set applied to the GERICO model.
